# Assessing the efficacy of a single-cycle measurement and adjustment strategy for direct oral anticoagulants in frail older patients: rationale and design of the DOAC-FRAIL randomized controlled trial

**DOI:** 10.1016/j.rpth.2026.106800

**Published:** 2026-06-17

**Authors:** Fabienne J.H. Magdelijns, Melanie J. de Jong, Kristien Winckers, Astrid D.H. Brys, Dionne C.W. Braeken, Sander M.J. van Kuijk, Henri M.H. Spronk, Hans Bosman, Daisy J.A. Janssen, Robin M.J.M. van Geel, Dave Hellenbrand, Hugo ten Cate, Yvonne Henskens

**Affiliations:** 1 Division of General Medicine, Department of Internal Medicine, Section of Geriatric Medicine, Maastricht University Medical Center+, Maastricht, the Netherlands; 2 The Cardiovascular Research Institute Maastricht School for Cardiovascular Disease, Maastricht University, Maastricht, the Netherlands; 3 Thrombosis Expert Center Maastricht, Maastricht University Medical Center+, Maastricht, the Netherlands; 4 Division of General Medicine, Department of Internal Medicine, Section of Vascular Medicine, Maastricht University Medical Center+, Maastricht, the Netherlands; 5 Department of Geriatrics, Ghent University Hospital, Ghent, Belgium; 6 Department of Clinical Epidemiology and Medical Technology Assessment, Maastricht University, Maastricht, the Netherlands; 7 CTD Netherlands, Dutch Thrombosis Services Client Council, Leiden, the Netherlands; 8 Department of Expertise and Treatment, Proteion, Horn, the Netherlands; 9 Department of Health Services Research and Department of Family Medicine, Care and Public Health Research Institute, Faculty of Health Medicine and Life Sciences, Maastricht University, Maastricht, the Netherlands; 10 Department of Clinical Pharmacy & Toxicology, Maastricht University Medical Center, Maastricht, the Netherlands; 11 Nutrition and Translational Research in Metabolism Institute of Nutrition and Translational Research in Metabolism, Maastricht University, Maastricht, the Netherlands; 12 Department of Clinical Chemistry, Maastricht University Medical Center+, Maastricht, the Netherlands

**Keywords:** anticoagulants, frail elderly, hemorrhage, thrombosis

## Abstract

****Background**:**

Frail older patients are at high risk of both thromboembolism and bleeding, making anticoagulant therapy challenging. Direct oral anticoagulants (DOACs) have largely replaced vitamin K antagonists because of comparable efficacy and lower bleeding risk. However, evidence supporting their use in frail older patients remains limited. Recent studies have demonstrated that a substantial proportion of frail older patients have DOAC plasma levels outside the expected on-therapy range, which may be associated with an increased risk of bleeding or thromboembolic events.

****Objectives**:**

The DOAC-FRAIL randomized controlled trial (RCT) aims to determine whether adjustment of DOAC therapy based on peak plasma concentrations improves the safety and efficacy of anticoagulant treatment compared with standard care in frail older patients.

****Methods**:**

The DOAC-FRAIL study is an international, multicenter, randomized controlled trial including frail older patients aged ≥70 years with a Clinical Frailty Scale score >3 receiving DOAC therapy. Participants are randomized to either a Single Cycle Measurement and Adjustment Strategy based on peak DOAC plasma concentrations or standard care. Patients with a life expectancy of less than 3 months or previous DOAC dose adjustment based on plasma level measurements are excluded. The primary outcome is the composite of bleeding events, including clinically relevant non-major bleeding, and thromboembolic events during 2 years of follow-up. Secondary outcomes include feasibility, determinants of deviant peak DOAC plasma concentrations, quality of life, cost-effectiveness, and all-cause mortality

****Results**:**

The DOAC-FRAIL trial is currently ongoing. Participant recruitment has been initiated, and study results are expected after completion of follow-up.

****Conclusion**:**

The DOAC-FRAIL RCT will provide evidence on whether a plasma concentration-guided DOAC dosing strategy improves clinical outcomes compared with standard care in frail older patients. The findings may contribute to more personalized anticoagulant therapy in this vulnerable population.

****Trial registration number**:**

EU CT-2025-521362-10-00.

## Essentials

• Frail older adults taking blood thinners have a high risk of both blood clots and bleeding, but it is unclear whether the current dose is optimal for every patient.
• The DOAC-FRAIL study will investigate whether measuring blood thinner levels and adjusting treatment when needed can improve care for frail older adults.
• The study will evaluate whether this personalized treatment approach can reduce bleeding and blood clot complications compared with standard care.
• The findings may support safer, more effective, and more personalized use of blood thinners in frail older adults.

## 1 Introduction

When considering anticoagulant therapy in frail older patients, balancing efficacy and bleeding risk is a challenge as this population is at high risk of both thromboembolisms, including ischemic stroke, and bleeding. Nowadays, direct oral anticoagulants (DOACs) have largely replaced vitamin K antagonists (VKAs) for major indications like atrial fibrillation (AF) after clinical trials showing that DOACs are noninferior to VKAs [[1], [2], [3], [4]]. In addition, DOACs are also increasingly prescribed to frail older patients as their bleeding risk appears more favorable than with VKA therapy, with lower rates of intracranial and fatal bleeding. Moreover, DOAC therapy does not require routine laboratory monitoring or frequent dose adjustments, making its use more practical.

Nonetheless, only limited evidence is available for DOAC use in the frail older population because of their poor representation in major randomized controlled trials (RCTs). In fact, a Dutch RCT showed an increased risk of major bleeding without an efficacy benefit in frail older patients with AF switched from VKA to DOAC [5]. One explanation for this increased risk of bleeding is the altered pharmacokinetics of DOACs in this population. In a French observational study, a substantial proportion of older patients had DOAC-plasma levels outside the expected on-therapy range [6], an observation that was confirmed in our prior studies [7,8] and others [9,10]. For example, in our DOAC-FRAIL pilot study (*N* = 42) among frail older patients, a deviant DOAC peak plasma level, defined as “outside the expected on-therapy range,” was found in 54% of patients during acute hospitalization and in 31% at the outpatient clinic [8]. In addition, we found the same results in our DOAC-FRAIL nursing home study in which 40% of nursing home residents had a deviant DOAC peak plasma level (unpublished data). Continuing from these results, the recent study of Godino et al. [11] revealed marked associations between high DOAC-plasma levels and higher odds of bleeding and low DOAC-plasma levels and higher odds of thrombosis in a frail older cohort, which confirms previous study results [12,13]. With this in mind and considering the aging population and the subsequent increase in DOAC use among frail older patients, including nursing home residents, for whom guideline-based recommendations are lacking regarding the management of DOAC treatment, it is crucial to evaluate the efficacy and safety of DOAC use in this specific population. Therefore, more research is needed in the vulnerable older population to facilitate appropriate and safe DOAC use in this group.

When considering all elements, we hypothesized that adjustment of DOAC use based on DOAC peak plasma level determination would reduce (in particular) the bleeding risks seen in the FRAIL-AF [5] and the 4Levels Study [11] in the (frail) older population compared with standard of care DOAC administration. Therefore, we aim in the DOAC-FRAIL RCT to evaluate the efficacy of a single-cycle measurement and adjustment strategy based on DOAC peak plasma levels in the frail older population (including nursing home residents). Within this strategy, DOAC use will be adjusted (either dose adjustment or switch to another DOAC) when a deviant DOAC peak plasma level is measured, aiming to reduce the incidence of (in particular) bleeding (including major and clinically relevant nonmajor [CRNM] bleeding) and thromboembolism (more as safety endpoint).

The aim of this article is to promote methodological transparency, facilitate critical appraisal of the study design prior to availability of results, reduce the risk of selective reporting, and support harmonization and replication of future research in this field. In addition, given the limited evidence currently available regarding DOAC monitoring in frail older adults, we believe that sharing the design and rationale of this study is of relevance to the clinical and scientific community.

## 2 Methods and Analysis

### 2.1 Study design and objectives

**Figure 1:**
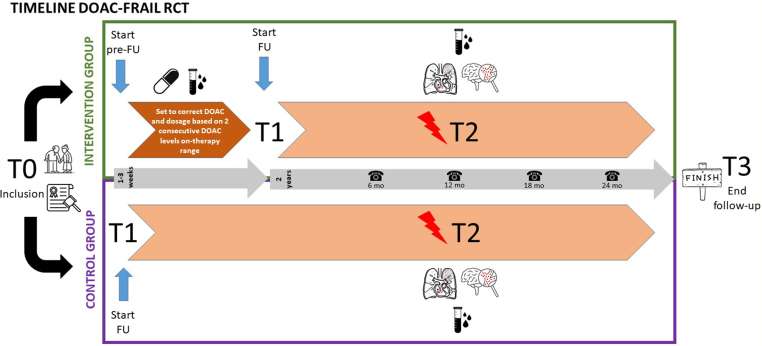
Timeline of the DOAC-FRAIL RCT, in which T0 represents the moment of inclusion, T1 the moment of bleeding or thromboembolism, and T2 the end of the study. DOAC, direct oral anticoagulant; FU, follow-up; RCT, randomized controlled trial.

The study is a 2-country, multicenter, prospective RCT. The primary objective is to evaluate the effect of a single-cycle measurement and dose adjustment strategy vs standard care. Specifically, we will investigate whether this strategy reduces the incidence of major and CRNM bleeding and thromboembolism in frail older patients (including nursing home residents). Secondary objectives are to investigate the feasibility of this single-cycle measurement and adjustment strategy, as well as the prevalence and determinants of deviant DOAC peak plasma levels in the frail older population, differences in quality of life, cost-effectiveness, and mortality between the intervention and control groups. Figure 1 shows the full timeline of the DOAC-FRAIL RCT in both arms.

### 2.2 Setting and patient population

Patients attending the outpatient clinic of the internal medicine departments, in particular, the geriatric and vascular medicine outpatient departments, and the cardiology outpatient departments of different (academic and regional) hospitals in the Netherlands and Belgium, and residents of nursing homes affiliated to the Limburg Living Lab in Ageing and Long-Term Care, are potentially eligible if they meet the inclusion criteria. The inclusion criteria are: (1) aged ≥ 70 years, (2) chronic DOAC use (for AF/venous thromboembolism), (3) frailty (defined as a Clinical Frailty Score [CFS] >3), (4) able to visit the outpatient clinic (only applicable to community-dwelling patients) and (5) has the capacity to provide informed consent. Exclusion criteria are a life expectancy of < 3 months estimated by the attending physician or prior adjustment of DOAC use based on DOAC level measurement.

In addition, hospitalized patients admitted to the departments of internal medicine and cardiology in the same hospitals are potentially eligible. However, participants included during their in-hospital stay will be randomized after a sufficient time of medical stability, defined as minimally 5 to 7 days after discharge.

Within the DOAC-FRAIL RCT, there will be a representation of both DOAC-naïve (defined as patients who had previously received a total of ≤ 2 months of DOAC therapy) and DOAC-experienced patients.

### 2.3 Sample size calculation

For the sample size calculation, we made the following assumptions. First, the risk reduction applies to the entire intervention group, as the intervention consists of a single-cycle measurement and adjustment strategy, irrespective of whether this leads to a dose adjustment or DOAC switch. Second, the yearly incidence of the composite endpoint is assumed to be 15% to 20% in the control group, based on the literature [14], and 10% to 15% in the intervention group, based on an estimated absolute reduction of 5% after the single-cycle measurement and adjustment strategy. With a 1:1 allocation and a 2-sided α level of 0.05, 686 to 905 participants are required in each treatment arm to have a power of at least 0.80. Considering a drop-out percentage of ∼10%, a total of 1509 to 1991 participants is required and will be included.

### 2.4 Study procedures

#### 2.4.1 Recruitment of participants and informed consent procedures

Participants will be recruited by their attending physician in the hospital, outpatient clinic, or nursing home. If an individual is deemed potentially eligible and expresses interest, they will be contacted by a member of the research team as soon as possible. During this contact, the participant will be verbally informed about the study, all questions regarding this study will be answered, and the participant will be provided with the relevant informational documents. After the participant has had sufficient time (a minimum of 7 days) to review the information, a follow-up call will be made to indicate whether the individual wishes to participate in the DOAC-FRAIL RCT. If the participant is willing to take part, the research team member will ask the participant to briefly summarize their understanding of the study to ensure they fully comprehend the nature of their involvement. After this confirmation, written informed consent will be obtained in the presence of a study team member. Informed consent will be obtained prior to any study related procedure being undertaken at screening. Informed consent will be written, dated, and signed by the person performing the interview and by the participant. The participant will be provided with a copy of the document by which informed consent has been given. Once the participant has been officially enrolled, a note will be added to their electronic patient record (EPR) to document their inclusion in the study.

#### 2.4.2 Randomization and blinding

After written informed consent has been given, the coordinating investigator will randomly assign the participant using Castor electronic data capture software. For in-hospital participants, the randomization will occur after a medically stable situation. As we would like to ensure a balance in sample size across the intervention and control groups, we will use block randomization. Randomization will additionally be stratified by center and by new vs “experienced” users. Blocks will be randomly chosen to determine the assignment of all participants.

Blinding of study participants is not feasible, nor is blinding for health care providers. However, blinding will be implemented for outcome assessors.

#### 2.4.3 Baseline data collection

In all patients, during the baseline visit, the following patient demographics and characteristics will be collected: medical history (Charlson Comorbidity Index [15]), weight, length, medication use, age, sex, frailty using the CFS [16,17], cognition using the Six-Item Cognitive Impairment Test [18], calf circumference, 4-m walk test and hand grip strength to screen for sarcopenia/low muscle mass, history of falling, Activities of Daily Living using the KATZ-score [19], Lawton Instrumental Activities of Daily Living score [20], living situation, CHA_2_DS_2_-VA (Congestive heart failure, Hypertension, Age, Diabetes, Stroke, Vascular disease, Age) and HAS-BLED (Hypertension, Abnormal renal/liver function, Stroke, Bleeding, Labile INR, Elderly, Drugs/alcohol) scores, and assessment of quality of life using the EQ-5D-5L questionnaire [21] (Supplementary Table 1). In addition, routine laboratory tests (creatinine and estimated glomerular filtration rate, hemoglobin and platelet counts, alanine transaminase and gamma-glutamyltransferase) will be performed. Information on bleeding and thrombosis history and medication adherence using the Medication Adherence Rating Scale 5 [22] will be collected.

#### 2.4.4 Intervention group

**Figure 2:**
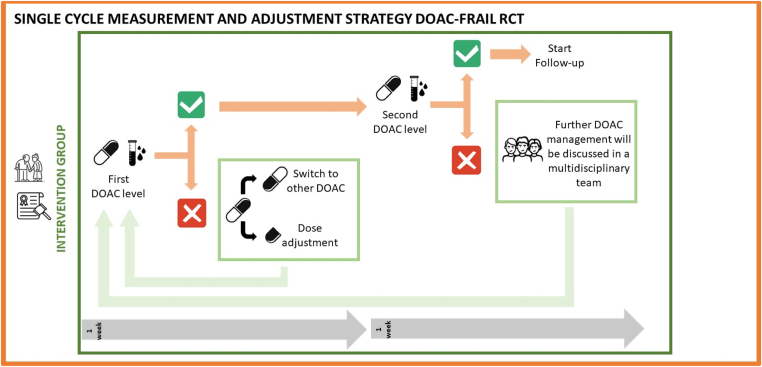
Timeline of our single-cycle measurement and adjustment strategy. DOAC, direct oral anticoagulant; RCT, randomized controlled trial.

*Single-cycle measurement and adjustment strategy.* In participants randomized to the intervention group, a single-cycle measurement and adjustment strategy will be applied. Participants will have their blood drawn as soon as possible after inclusion (but no earlier than 3 days after initiation of a DOAC) to measure a first peak DOAC level (2-4 hours after intake). The following scenarios can occur (Figure 2):

(1) First peak DOAC level is within the on-therapy range → a second peak DOAC level will be measured 1 week later → the second peak DOAC level is again within the on-therapy range → no adjustment is necessary, and participant continues to follow-up.
(2) First peak DOAC level is outside the on-therapy range → dose adjustment or switch to another DOAC if dose adjustment within an approved dose range is not possible → a new first peak DOAC level will be measured 1 week later, after which 1 of the 3 scenarios described will be reassessed. This process could potentially continue indefinitely; however, 2 primary rules will be applied—except in the case of rivaroxaban (Supplementary Figures), as literature indicates a higher incidence of bleeding complications are associated with its use. The 2 primary rules are as follows: (1) dosage adjustments are permitted only once, and (2) switching to an alternative DOAC is allowed only once. For each DOAC, including each specific dose, a Standard Operating Procedure (SOP) has been established (Supplementary Figures). These SOPs include a flowchart outlining the adjustments to be made based on specific DOAC levels.
(3) First peak DOAC level is within the on-therapy range → a second peak DOAC level will be measured 1 week later → the second peak DOAC level is outside the on-therapy range → further DOAC management will be discussed in a multidisciplinary team consisting of experts in anticoagulant management.

Based on the literature (Beers criteria [23]), we have specified apixaban and edoxaban as our first-choice DOACs in our SOPs. In circumstances in which initiating a participant on a DOAC is unsuccessful, ie, a participant has deviant DOAC levels after an adjustment has been made and after an individual treatment plan of the multidisciplinary team has failed, we will recommend the patient to switch to a VKA.

Importantly, in cases in which the DOAC level is not measurable or very low, we will first address therapy compliance with the participant, and a second DOAC level measurement will be performed before considering dose adjustment or switching to another DOAC.

*DOAC level measurement.* Blood sample analysis will be performed using commercially available and validated tests. For the measurement of DOAC levels, the following procedure will be followed. First, verification will be carried out to ensure that the DOAC was taken 2 to 4 hours prior to the blood sample collection. Blood will be drawn with minimal stasis in a 3.2% citrated vacuum tube (Becton Dickinson) via a puncture of the antecubital vein. Blood will be centrifuged in a 2-step procedure. First, it will be centrifuged for 5 minutes at 2500*g* at room temperature. For the second step, plasma will be centrifuged for 10 minutes at 10,000*g* at 18 °C. The Diluted TT Hemoclot test will be used for the measurement of dabigatran levels, and activated factor X (FXa) activity for the measurement of apixaban, rivaroxaban, and edoxaban levels (both Hyphen Biomed). More in detail, we will be using a clotting activity assay on the CS2500 Analyzer (Sysmex/Siemens), which employs a chromogenic substrate. This assay is the most widely used method globally to measure DOAC levels. It is ISO-15189 certified and registered under the In Vitro Diagnostic Regulation. This anti-FXa assay measures the effect of the DOAC and its active metabolites in relation to an excess FXa. The chromogenic substrate in the assay is cleaved by the residual FXa. Variations in active metabolites, which may be influenced by factors such as renal function disorders, are also assessed through this activity assay. Any shifts in the metabolite ratio will be reflected in the FXa activity measurement.

Dabigatran, apixaban, rivaroxaban, and edoxaban calibrators and assay controls will be obtained from Hyphen Biomed. All measurements will be performed according to the manufacturer’s instructions without modifications. Further, time from the last DOAC intake to time of blood draw will be recorded and, to interpret the DOAC level, we will use expected on-therapy ranges for peak plasma levels, defined as DOAC level measured within 2 to 4 hours after intake.

*Rationale for the use of DOAC peak plasma levels.* In the present study, we have opted to base our single-cycle measurement and adjustment strategy on DOAC peak plasma levels for several reasons. First, given our objective to reduce complications associated with DOAC use in the frail older population—who are particularly susceptible to bleeding risks, as shown by studies such as the FRAIL-AF trial [5]—deviant DOAC peak plasma levels are closely related to the risk of bleeding and thrombosis [11,12]. Second, as one of the primary advantages of DOACs is the absence of routine monitoring requirements, we sought to design a strategy that minimizes the need for intensive monitoring. As a result, we have chosen to measure only 1 DOAC level (peak), rather than both trough and peak plasma levels. This approach not only simplifies the strategy but also reduces the burden on patients, as it requires only a single blood sample. Finally, by limiting the strategy to peak-level measurement, we are able to reduce associated costs, further enhancing the feasibility of our strategy.

*DOAC peak-level interpretation.* For the DOAC peak-level interpretation, we will use the data (expected on-therapy ranges) recommended in the antithrombotic guidelines of the Federatie Medisch Specialisten in the Netherlands [24]. These expected on-therapy ranges are in line with data published by Douxfils et al. [25]. The Table shows the expected therapeutic ranges of peak DOAC levels as we will use them in the DOAC-FRAIL RCT.

**Table:** Expected on-therapy ranges of peak DOAC levels for atrial fibrillation and venous thromboembolism.

DOAC		Tmax (h)	Expected range	
			Lower limit (ng/mL)	Upper limit (ng/mL)
Apixaban, 5 mg	2 times daily	3-4	59	302
Apixaban, 2.5 mg	2 times daily	3-4	30	153
Edoxaban, 60 mg	1 time daily	1-2	125^a^	317^a^
Edoxaban, 30 mg	1 time daily	1-2	55^a^	225^a^
Rivaroxaban, 20 mg	1 time daily	2-4	177	361
Rivaroxaban, 15 mg	1 time daily	2-4	134^b^	278^b^
Rivaroxaban, 10 mg	1 time daily	2-4	91	195
Dabigatran, 150 mg	2 times daily	0.5-2	60	450
Dabigatran, 110 mg	2 times daily	0.5-2	80	300

All expected on-therapy ranges are based on data published in the antithrombotic guidelines of the Federatie Medisch Specialisten in the Netherlands. They are in line with data published by Douxfils et al. [25].

DOAC, direct oral anticoagulant.

a As no data is available on the expected therapeutic ranges for edoxaban, we will use the expected therapeutic ranges established by the laboratory of Maastricht University Medical Center (MUMC+).

b Since there is no available data on the expected range for rivaroxaban 15 mg, we will use the average of the therapeutic ranges for rivaroxaban 20 mg and 10 mg.

#### 2.4.5 Control group

In participants randomized to the control group, standard care will be given. Standard care includes that the choice of type and dose of DOAC are left at the discretion of the treating physician. There will be no interference in the choice or dose of DOAC at study inclusion. Further, it is up to the attending physician to follow guidelines on follow-up (renal function, body weight, etc.), but we assume this will at least include the annual check.

Additionally, in a subgroup of participants (all participants included in the MUMC+) blood samples will be collected at the time of inclusion and stored for future analyses.

#### 2.4.6 Assessments of study outcome(s)

For the primary outcome, bleeding will be assessed using the International Society on Thrombosis and Haemostasis Bleeding Assessment Tool [26], of which a modified version will be used in the follow-up visits. Thrombosis, more as safety endpoint, will be assessed through history taking. In addition, the EPR will be evaluated for adverse events.

For certain secondary objectives, the following questionnaires will be used as outcomes: EQ-5D-5L score, KATZ scale, and Lawton & Brody scale. Feasibility will be assessed through a qualitative study, including semistructured interviews with health care professionals and patients. Other secondary outcomes will be collected by anamnesis during the follow-up visits and reviewing the EPR (determinants of deviant DOAC levels, mortality). Finally, a cost analysis will be conducted to assess the balance between the additional costs associated with this strategy and the health care–related cost savings it may prevent.

**Figure 3:**
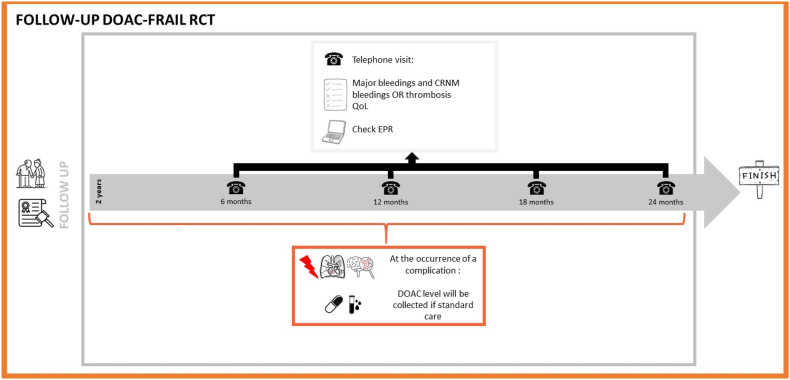
Timeline of the follow-up of the DOAC-FRAIL RCT. CRNM, clinically relevant nonmajor bleeding; EPR, electronic patient record; DOAC, direct oral anticoagulant; RCT, randomized controlled trial.

#### 2.4.7 Follow-up visits

During the 2-year follow-up period of the study, participants will be contacted every 6 months by telephone (Figure 3).

During the telephone visit, the occurrence of bleeding or thrombosis will be evaluated and several questionnaires, as stated above, will be completed. Telephone visits are preferably conducted with the participant; alternatively, they may be conducted with a family member if necessary. For nursing home residents unable to complete questionnaires, a health care professional is contacted to assess, eg, occurrence of bleeding. In addition to these telephone visits, the EPR system will be consulted to identify/confirm the occurrence of complications and evaluate, eg, compliance.

Information on DOAC levels measured at the occurrence of a complication (bleeding/thrombosis) in the total research population will be collected through evaluation of the EPR. Nowadays, it is often standard care to perform a DOAC level measurement in such urgent medical situations in many academic hospitals.

In addition, evaluation of the EPR will be conducted to identify any (potentially) missing data and to gather information on changes in kidney function, medication, weight, and the standard care provided.

### 2.5 Data analysis

After the first 100 patients in the intervention group have completed the single-cycle measurement and adjustment strategy, an analysis will be conducted to determine whether a second DOAC measurement is necessary, provided that the first DOAC measurement falls within the expected therapeutic range.

Baseline characteristics will be presented stratified by allocation. The primary analysis will be performed according to the intention-to-treat principle. In addition, a per-protocol analysis will be conducted. To evaluate whether the single-cycle measurement and adjustment strategy is superior to standard care with respect to the composite endpoint, a competing risks survival model according to Fine and Gray will be used to analyze time to first event, accounting for competing events. The effect of treatment will be expressed as hazard ratios with corresponding 95% CIs. The proportional subdistribution hazards assumption will be assessed using appropriate diagnostic methods. Recurrent events and their nature will be summarized descriptively. Depending on the distribution of event counts, additional analyses using Poisson regression may be performed to formally test differences in the number of events between groups.

Furthermore, the aforementioned analyses will also be conducted separately for new users and long-term users. In addition, subgroup analyses in participants aged > 70, > 80, and > 90 years will be performed. Last, participants requiring a switch to VKA will be excluded to perform subgroup analyses to evaluate the effect in participants who use a DOAC.

Two-sided *P* values < .05 will be regarded as statistically significant. Missing data will be imputed depending on the extent of missing data and the likely mechanism. Results will be expressed as odds/hazard ratios including 95% CIs.

Descriptive statistics will be used to present the prevalence of deviant DOAC levels. Logistic and linear regression analyses will be performed to estimate associations between potential predictors of deviant DOAC levels and actual levels (linear model) and a deviance indicator (logistic model). Mixed-effects regression models for repeated measures will be used to assess the effect of the intervention on quality of life over the course of follow-up. Fixed effects will be time, study group, their interaction, and a set of potential confounding variables.

The feasibility and acceptability of the single-cycle measurement and adjustment strategy will be evaluated within a qualitative study. A cost–utility study will be performed to evaluate the cost-effectiveness of our intervention. Health care consumption will be collected from the EPRs and linked to Dutch unit costs. Kaplan–Meier survival curves will be used to visually represent the survival experience of participants in the 2 groups. To formally test differences in survival rates, Cox proportional hazards regression will be performed, adjusted for potential confounders (such as age, sex, and comorbidities).

Additional explorative analyses will be performed after stratification for eg, DOAC, new users vs experienced users, and adjustments due to DOAC peak plasma levels above and below the expected on-therapy range.

### 2.6 Patient involvement

A patient representative, who is also a member of the Client Council Thrombosis Services in the Netherlands, is part of the DOAC-FRAIL study team. He played an important role in the conceptualization of the study protocol and will be involved during the duration of the study.

### 2.7 Ethics

The DOAC-FRAIL RCT will be conducted according to the principles of the Declaration of Helsinki (last revision, October 2024) and in accordance with the Dutch Medical Research Involving Human Participants Act law (WMO). The study protocol was approved by the Medical Research Ethics Committee (MREC) of MUMC+ (reviewing committee) and by the Central Committee on Research Involving Human Subjects (CCMO), the competent authority in the Netherlands (registration number EU CT-2025-521362-10-00). A Data Safety Monitoring Board has been established to oversee the study.

## 3 Discussion

The DOAC-FRAIL RCT will be the first study to evaluate the efficacy of a single-cycle measurement and adjustment strategy based on DOAC peak plasma levels in reducing the number of adverse events among frail older patients, including nursing home residents. In addition, the DOAC-FRAIL RCT will yield substantive, currently missing, data on the overall efficacy and safety, including tolerance and adherence of DOAC use in the frail older population.

Frail older patients have been underrepresented in the pivotal landmark trials of DOACs [[1], [2], [3], [4]]. Nevertheless, the evidence derived from these trials is commonly extrapolated to clinical practice in this population. Emerging data, however, suggest that DOACs should be prescribed with greater caution in frail older patients, highlighting the need for further research. For example, the FRAIL-AF study demonstrated that actively switching frail older patients from VKAs to DOACs was associated with an increased risk of bleeding (odds ratio, 1.69; 95% CI, 1.23-2.32) without an increase in thromboembolic events [5]. In addition, several studies have shown that DOAC levels in frail older patients are often outside the expected on-therapy range, both in acute and stable medical conditions [6,[8], [9], [10]]. The clinical consequence might be the occurrence of more than predicted numbers of adverse events, including thromboembolic events or bleedings, based on the repeatedly reported risk associations between high DOAC levels and bleedings and low DOAC levels and thromboembolisms [[11], [12], [13]]. The DOAC-FRAIL RCT will determine whether a single-cycle measurement and adjustment strategy may contribute to a further reduction of adverse events in the frail older population.

Important features of the DOAC-FRAIL RCT include the use of DOAC peak plasma levels in daily practice, the inclusion of DOAC-naïve as well as DOAC-experienced patients, and specific representation of frail (CFS >3) older (≥ 70 years) patients, including nursing home residents. An innovative element is that the study protocol provides guidance on the interpretation and management, based on deviant DOAC levels for each specific DOAC and dose. This way, for the first time, we implement this laboratory biomarker in the management of anticoagulant treatment in complex patients. Moreover, it is the first study to implement laboratory guidance through DOAC monitoring in any patient population.

A limitation of correlating DOAC-plasma levels with outcomes is that measured plasma levels reflect only a single time point and may not represent overall exposure or the balance between thrombotic and bleeding risk. In addition, DOAC-plasma levels are influenced by factors including renal function, comorbidities, medication use, adherence, sampling time, and illness, and the absence of therapeutic ranges complicates interpretation. Moreover, clinical outcomes are often multifactorial in origin. Nevertheless, frail older adults more often have DOAC-plasma levels outside expected ranges, which are shown to be independently associated with bleeding and thrombotic events. Maintaining DOAC-plasma levels within expected ranges may therefore help to reduce complications and support individualized anticoagulant therapy.

If our hypothesis is supported by the study outcomes, our single-cycle measurement and adjustment strategy will contribute to a reduction in bleedings (including CRNM bleedings) and/or thromboembolism in the frail older population. This would be a breakthrough in DOAC management in this population. In addition, it may improve safe switching from VKA to DOAC, in those that qualify for DOAC management, specifically in the frail older population. If the study hypothesis is refuted, the obtained study data will be helpful in further refining anticoagulant management, as it provides a basis for exploring additional risk factors in frail older adults, an understudied population.

## 4 Conclusion

This will be the first study to evaluate the efficacy of a single-cycle measurement and adjustment strategy for DOACs in frail older patients, including nursing home residents, in reducing adverse events, defined as major bleedings, CRNM bleedings, and thrombosis.
