# Supplementary material for: Assessing the efficacy of a single-cycle measurement and adjustment strategy for direct oral anticoagulants in frail older patients: rationale and design of the DOAC-FRAIL randomized controlled trial
Source: Res Pract Thromb Haemost. 2026 Jun 17;10(4):106800. doi: 10.1016/j.rpth.2026.106800 (PMC13380038; doi:10.1016/j.rpth.2026.106800)

**SUPPLEMENTARY MATERIAL**

Supplementary table 1

| Overview of measurements in the DOAC-FRAIL RCT | | | | |
| --- | --- | --- | --- | --- |
| *Measurements* | *Intervention group* | | *Control group* | |
|  | *Baseline* | *Follow-up* | *Baseline* | *Follow-up* |
| Medical history | X |  | X |  |
| Medication | X | X | X | X |
| Weight | X | X | X | X |
| Length | X |  | X |  |
| Age | X |  | X |  |
| Sex | X |  | X |  |
| Living situation | X | X | X | X |
| DOAC level | X |  | * |  |
| Additional blood (kidney function, hemoglobin, platelet count) | X | ^+^ | * | ^+^ |
| DNA (blood) | X |  | * |  |
| Bleeding (major and CRNM) using the Bleeding Assessment Tool (BAT) | X | X | X | X |
| Thrombosis (verbal evaluation) | X | X | X | X |
| Medication Adherence Rating Scale (MARS-5) | X | X | X | X |
| EQ-5D-5L | X | X | X | X |
| KATZ | X | X | X | X |
| Lawton & Brody scale | X | X | X | X |
| Six-Item Cognitive Impairment Test (6CIT) | X | X | X | X |
| Clinical Frailty Scale (CFS) | X | X | X | X |
| Calf circumference | X |  | X |  |
| 4 meter walk test | X |  | X |  |
| Hand Grip Strength | X |  | X |  |
| *Abbreviations: DNA Desoxyribonucleïnezuur, CRNM Clinically Relevant Non Major.*  **In the subgroup of participants included in the MUMC blood will be collected at baseline, but not evaluated prospectively.*  *^+^During follow-up visits, the electronic patient record will be reviewed to assess recent kidney function, hemoglobin levels, and platelet counts.* | | | | |

Supplementary figures


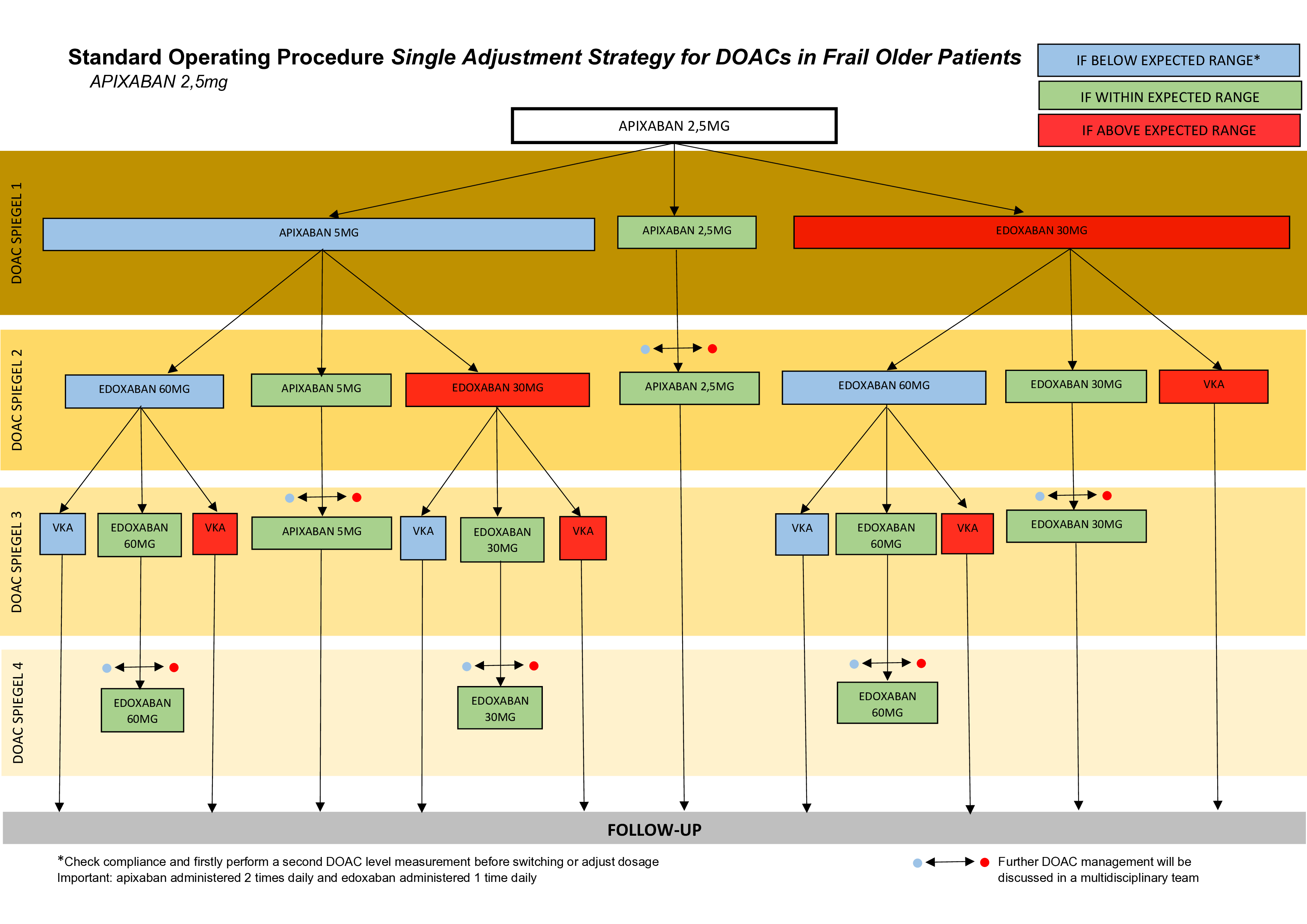


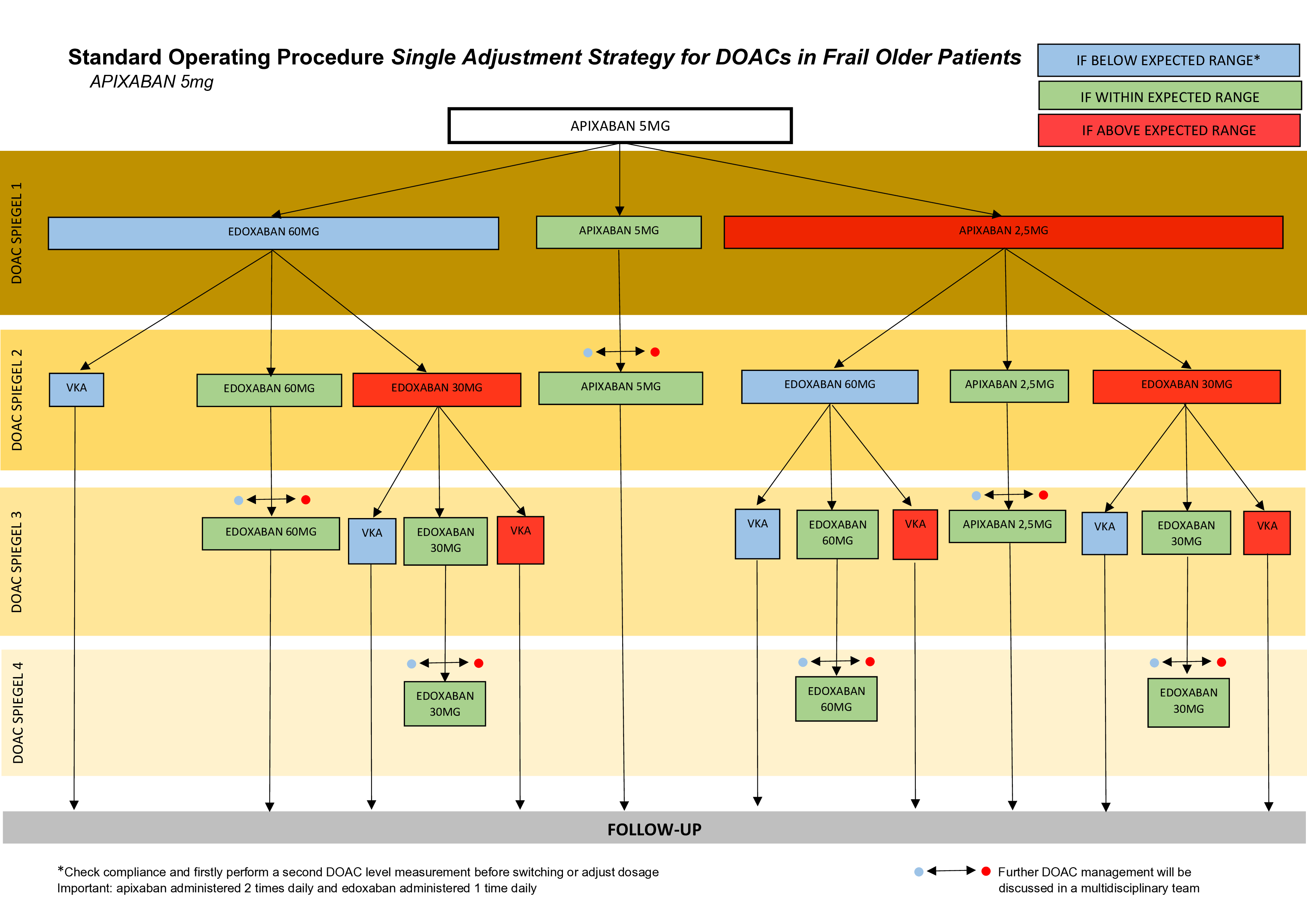


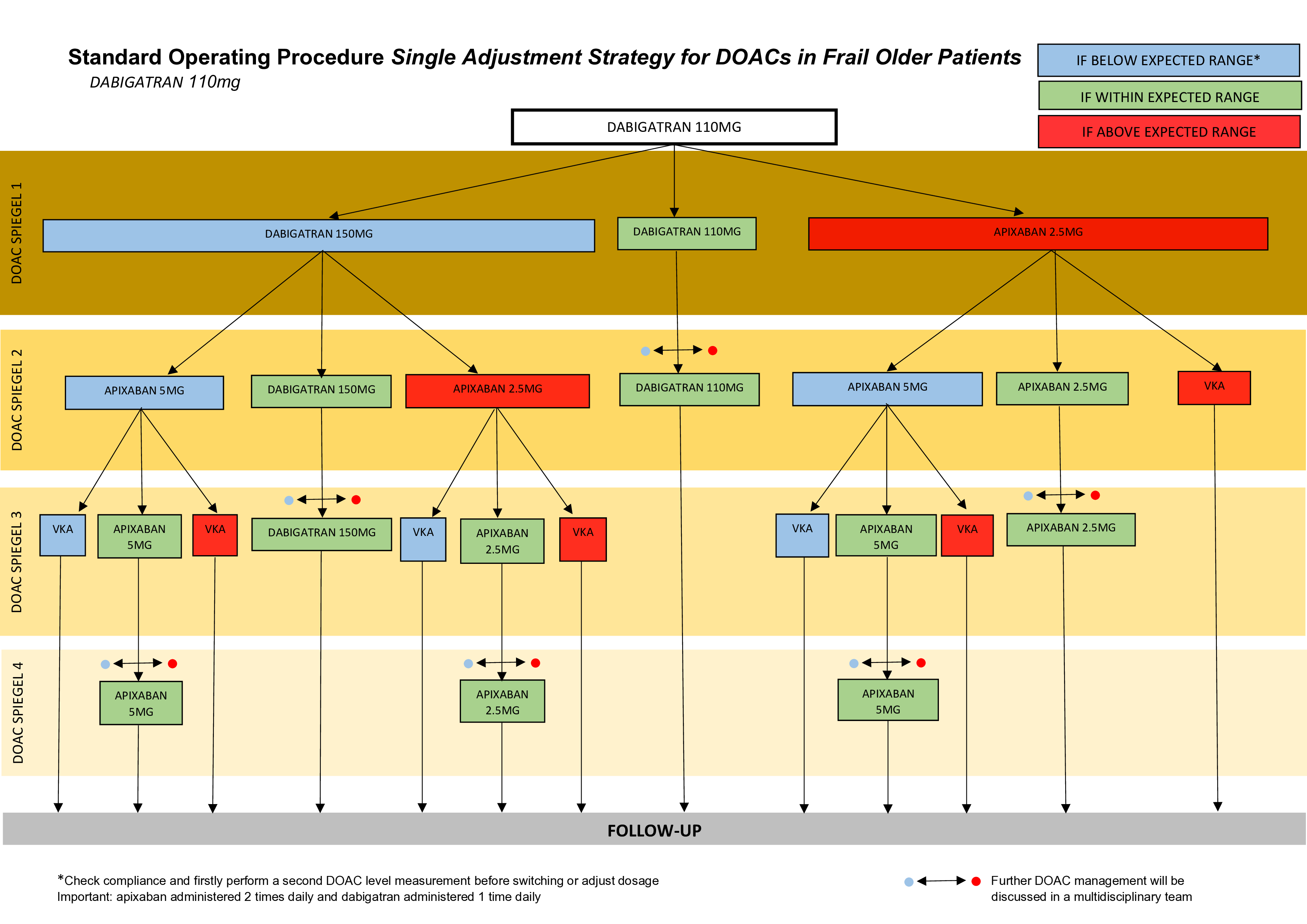


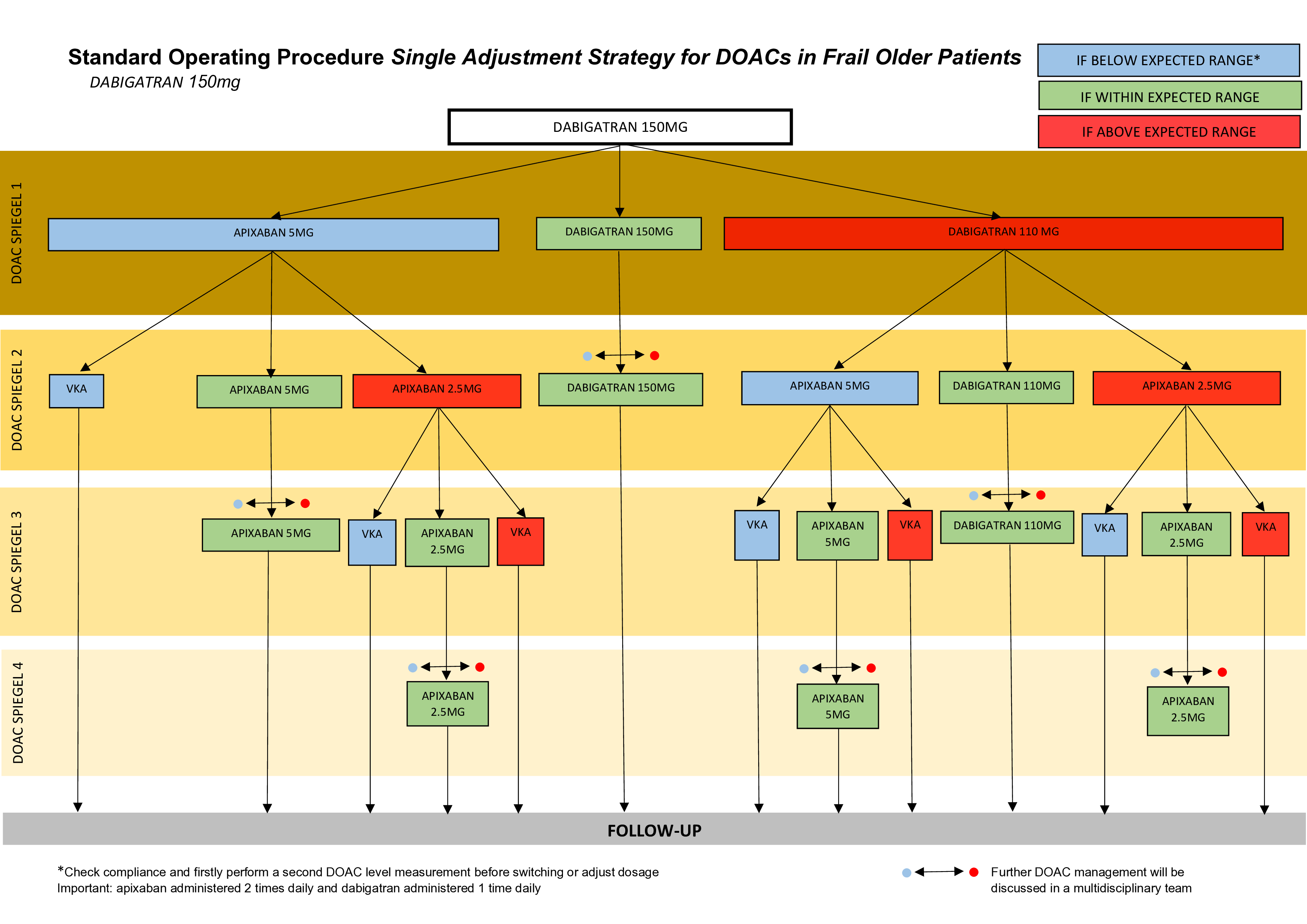


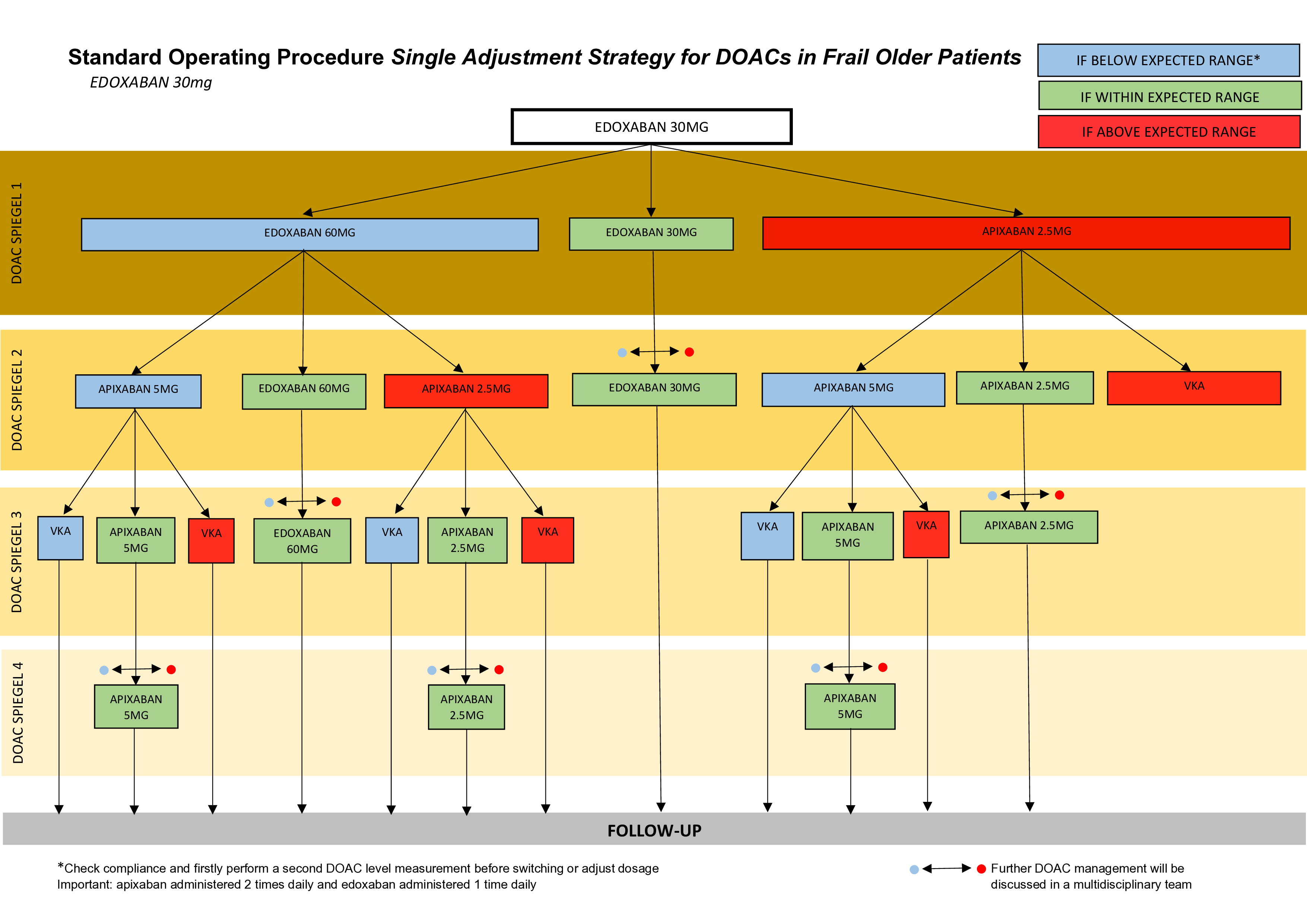


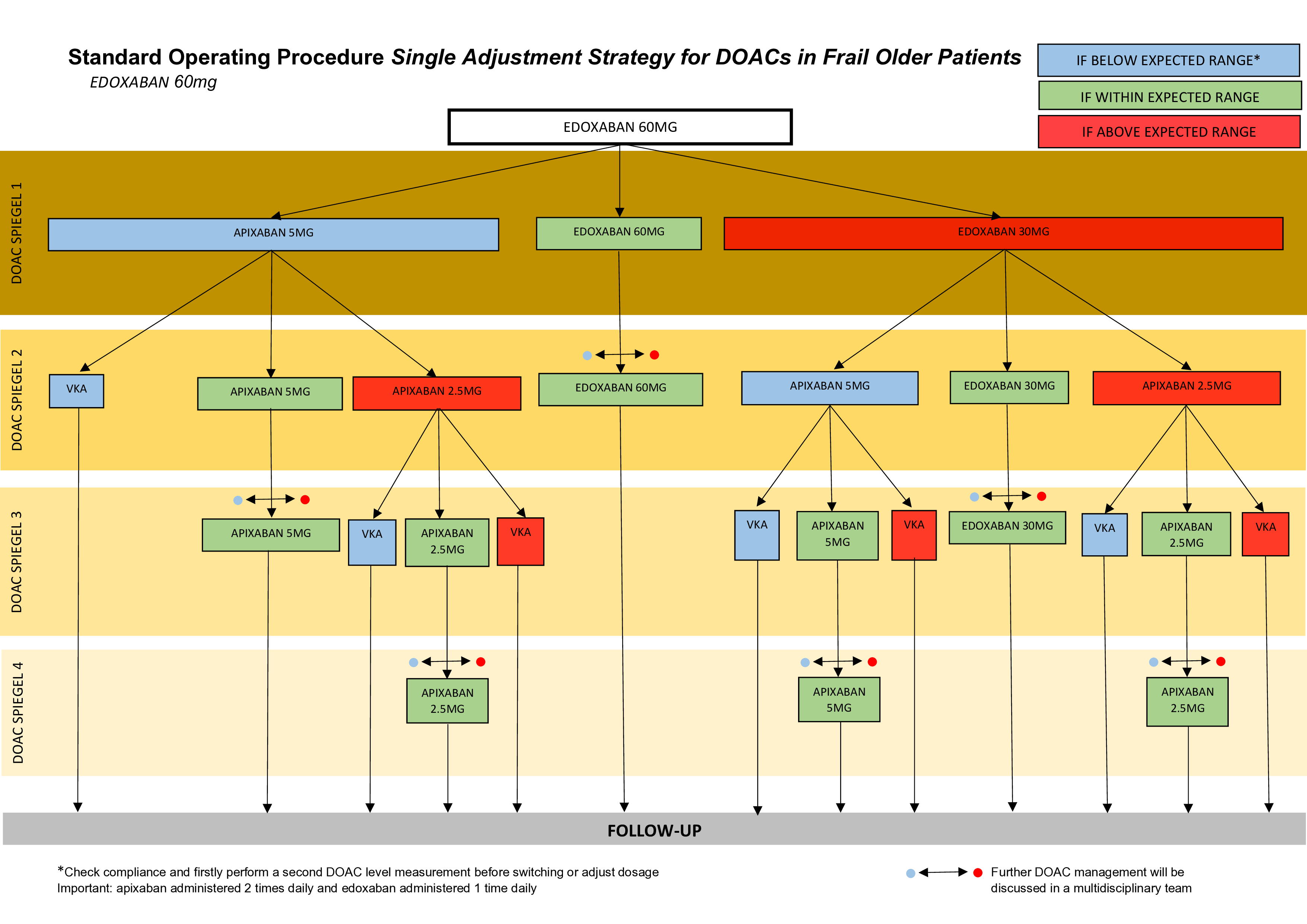


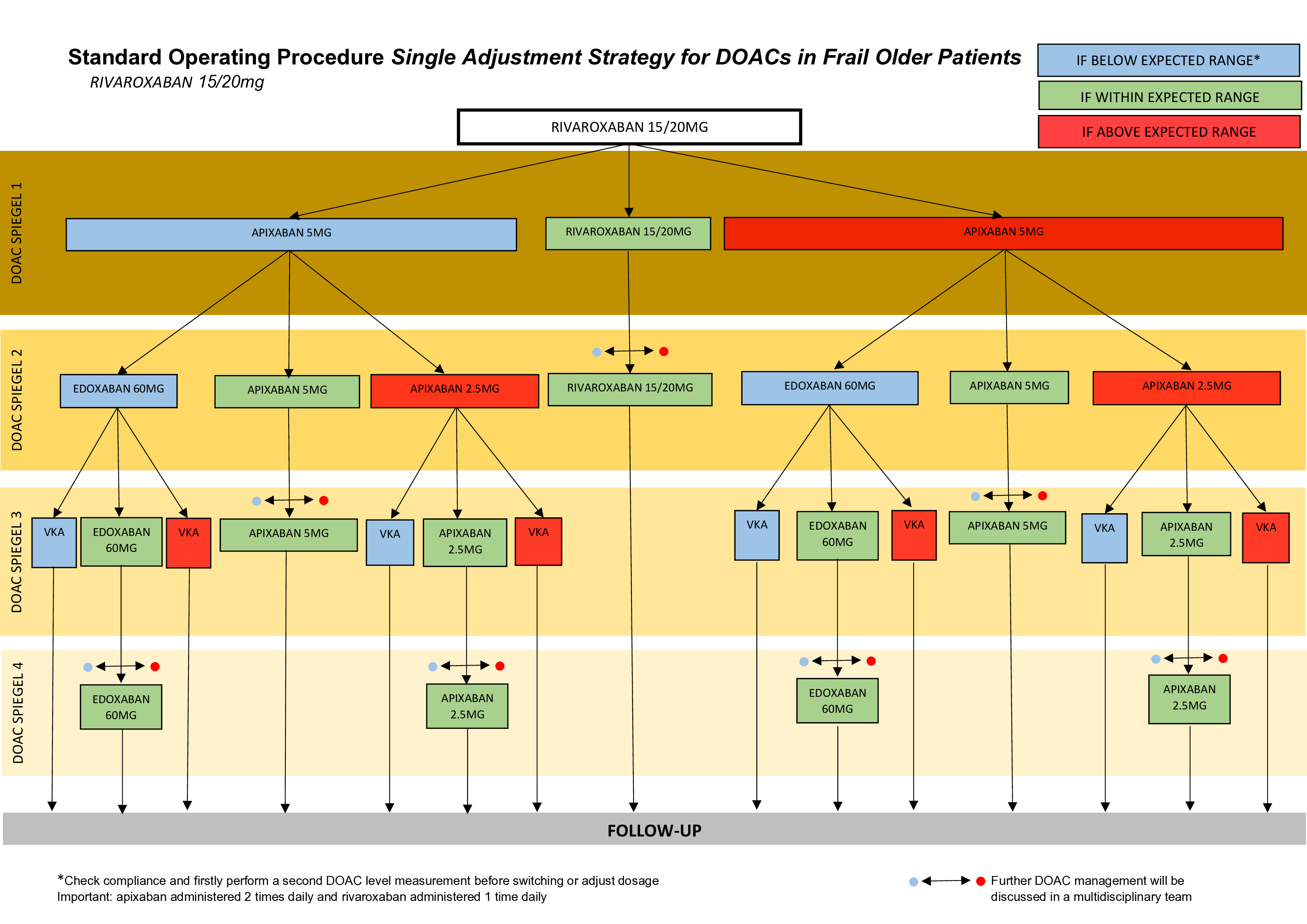

Supplement: Supplemental Table 1 [file mmc1.docx]
